# Use of Multi-Locus Metabarcoding to Inform an Australian Government Biosecurity Response on the Origins of Suspected Illegal Plant Products

**DOI:** 10.3390/ijms26115399

**Published:** 2025-06-04

**Authors:** Jennifer A. Soroka, Matias Silva-Campos, Frank Bedon, Adrian Dinsdale, Dianne M. Gleeson, Alejandro Trujillo-González

**Affiliations:** 1 Centre for Conservation Ecology and Genomics, Institute for Applied Ecology, University of Canberra, Canberra, ACT 2617, Australia; jenn.soroka@canberra.edu.au (J.A.S.); dianne.gleeson@canberra.edu.au (D.M.G.); 2 Australian Government Department of Agriculture, Fisheries and Forestry-Plant Innovation Centre Facility, Mickleham, VIC 3064, Australia; matias.silvacampos@aff.gov.au (M.S.-C.); frank.bedon@aff.gov.au (F.B.); adrian.dinsdale@aff.gov.au (A.D.); 3 Department of Ecological Plant and Animal Sciences, School of Agriculture, Biomedicine and Environment, La Trobe University, Bundoora, VIC 3086, Australia

**Keywords:** high-throughput sequencing, molecular testing, biosecurity response

## Abstract

Biosecurity is vital to Australia’s efforts to prevent and respond to pests and diseases. Here, we report on testing suspected illegal goods (SIGs) as part of an active Australian biosecurity response in Sydney. The Australian Government, Department of Agriculture, Fisheries and Forestry detected and secured consignments containing tuber products of unknown biosecurity risk and origin. Swab samples were collected from vacuum-sealed yam products, organic packing material (background negative controls), and field negative controls to assess possible cross-contamination from the storage facility. DNA from all samples was analysed using high-throughput metabarcoding targeting the Internal Transcribed Spacer 2 (ITS2) and the chloroplast *trnL* (UAA) P6 Loop gene regions by two independent teams in Australia. A plant community profile comprising Australian native species and other non-native established species would support the notion of produce being harvested and/or packaged domestically, while their absence would suggest foreign production. Of the 5,764,942 total reads produced, the bioinformatic analysis generated 5,181,530 amplicon sequencing variants employed for species identification. Twenty plant taxa were identified via ITS2 and 15 via *trnL*, corresponding to worldwide distributed plants, non-native species established in Australia, or species not recorded in Australia. No Australian endemic species were detected. The absence of common Australian native plants, combined with the presence of species not known to occur in Australia, provided strong evidence that the suspect tuber products were illegally imported.

## 1. Introduction

Maritime shipping is the backbone of global trade and integral to the international economy, with over 80% of global commodities being traded via shipping lanes [1]. In 2021, dry cargo (containerized trade, general cargo, and other minor bulk commodities) accounted for ~4.7 billion tons of transported product [2]. The shipping container trade is also the dominant mode of transportation of illicit goods including wildlife and livestock trade, pharmaceuticals, and human trafficking [3,4]. Illegal trade has intensified in recent years, with the black-market capitalizing on vulnerabilities generated by world crises [5]. Australia, for example, is a country where nearly all international trade passes through ports, along with a quarter of its domestic freight. As such, it is a country with strong biosecurity measures that play a critical role in how Australia prevents, responds to, and recovers from serious pests and diseases. Measures include scientific-based risk assessments for imported cargo risk profiling, where inspection for biosecurity control and decision on further action (e.g., treatment, isolation, holding) is determined by the Australian Government Department of Agriculture, Fisheries and Forestry (the Department).

On 15 February 2023, biosecurity officers from the Department made one of the largest biosecurity seizures in Australian history. More than 38 tonnes of potential biosecurity risk materials were seized including various plant products and 116 types of animal products such as turtles, frog legs, beef, pig, and avian meats [6,7]. To ascertain the origins of the intercepted plant produce, an integrated approach of conventional diagnostic methods as well as novel molecular and surveillance technologies was deployed to assess the associated biosecurity risk. Amongst the many methods used, environmental DNA-based (eDNA) metabarcoding was used to target specific genetic markers to examine the diversity of plant DNA associated with the illegal plant produce consignments.

The use of eDNA and molecular detection techniques are now being routinely applied in various fields including produce traceability and biosecurity surveillance [5,8]. For example, this technology has been successfully applied to perishable food, including honey, where pollen metabarcoding identifies floral sources and authenticates regional origins [9,10,11]. For surveillance, metabarcoding of eDNA, coupled with high-throughput sequencing (HTS), can be used to rapidly detect and identify single and multiple pest species in large-scale biodiversity assessment without the need for visual confirmation or extensive taxonomic knowledge [12]. These technologies have been beneficial in border surveillance of novel invasive pests, incursion responses, and in tracking population levels of established pests, targeting alien plants, plant pathogens, hitchhiker insects, and marine organisms, among others [13,14,15].

The Australian National eDNA Reference Centre (NeRC) collaborated with the department’s Plant Innovation Centre (PIC) to ascertain the origin of the plant produce in the seized consignment using eDNA-based molecular analyses to provide inference on plant species composition. Libraries were prepared from trace eDNA collected from tuber produce in seized pallets. Multi-locus plant metabarcoding analysis was conducted with amplified fragments from the nuclear ribosomal Internal Transcribed Spacer 2 (ITS2) (~226 bp) and the chloroplast *trnL* (UAA) intron P6 Loop (hereafter referred to as *trnL*) (~40–182 bp) [16].

## 2. Results

### 2.1. Sequencing Results

**Figure 1:**
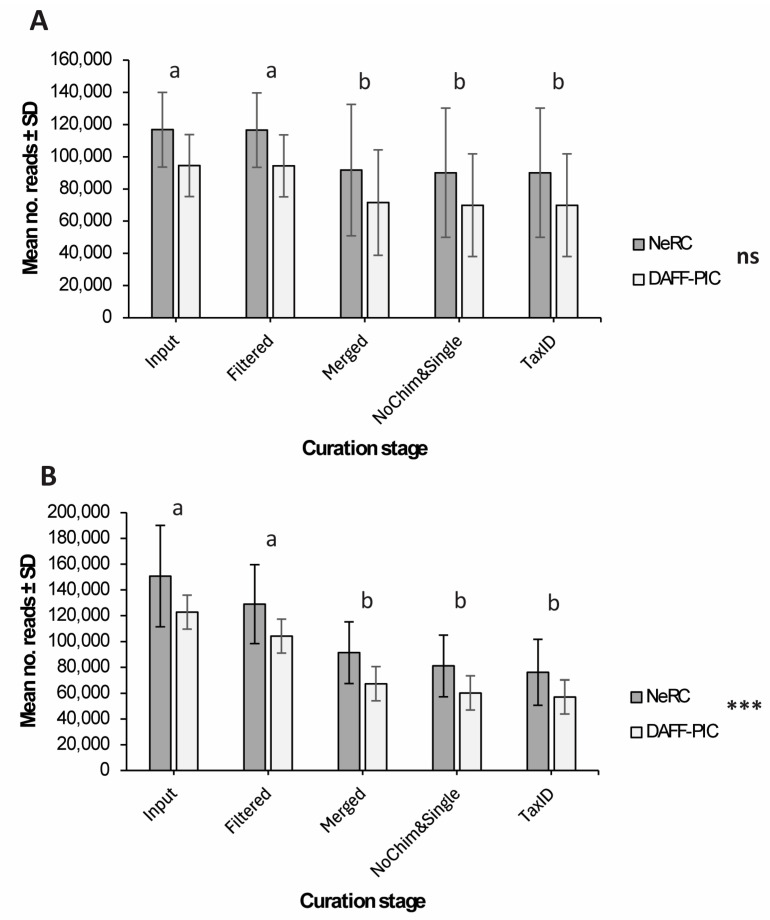
Mean number of curated reads ± Standard deviation obtained for the *trnL* (UAA) intron P6 Loop gene region (**A**) and the Internal Transcribed Spacer 2 region (**B**) across different stages of high-throughput sequencing curation. “Input” = Average number of reads per sample prior to processing; “Filtered” = average number of reads following curation for amplicon length and quality; “Merged” = average number of reads following pairing of forward and reverse reads; “NoChim&Single” = average number of reads following removal of chimeric amplicons and singleton Amplicon Specific Variances (ASVs); “TaxID” = average number of ASVs following taxonomic assessment. Letters “a” and “b” indicate post hoc Tukey HDS groups for significant differences across curation stages for each marker region. “ns” = no significant difference in number of reads achieved by each team throughout curation; “***” = there are significant differences in the number of reads achieved by each team.

High-throughput sequenced samples yielded a total of 3,976,979 and 5,181,609 reads for *trnL* and ITS2, respectively, across both teams (Supplementary S1, trackseq tabs). There was a significant loss of reads through the curation process across both teams for both *trnL* (Two-way ANOVA, F_4,170_ = 6.913, *p* < 0.001, Figure 1A) and ITS2 (Two-way ANOVA, F_4,170_ = 43.18, *p* < 0.001, Figure 1B). Following curation, samples prepared by NeRC produced an average of 95,379 ± 33,278 S.D reads for *trnL* (Figure 1A) and 76,092 (± 25,540 S.D) for ITS2 (Figure 1B) for which a taxonomic identity was achieved. Similarly, samples processed by PIC yielded an average of 67,434 ± 31,153 S.D reads for *trnL* (Figure 1A) and 51,641 ± 30,050 S.D reads for ITS2 (Figure 1B). There were no significant differences in the number of reads achieved by each team during curation for *trnL* (Two-way ANOVA, Figure 1B); however, the mean number of reads achieved by PIC for ITS2 was significantly lower than those produced by NeRC (Two-way ANOVA, F_1,170_ = 30.57, *p* < 00.1, Figure 1B).

### 2.2. Detections in Field Background and Negative Controls

**Figure 2:**
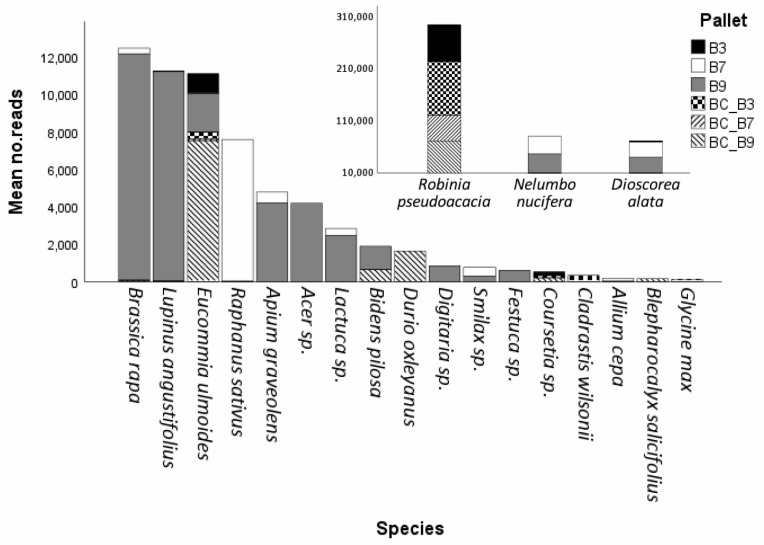
Mean number of reads for species detected across three pallets of biosecurity concern (B3, B7, and B9) and associated blank controls (BCs) across both gene targets and teams. Inlay graph shows the number of reads for species with detections with >10,000 reads.

Field background and negative controls showed a significantly high amplification of the plant species *Robinia pseudoacacia* (Fabaceae) (Two-way ANOVA, F_20,1093_ = 38.949, *p* < 0.001, Figure 2). Detections for this species were significantly high in pallet B3 (mean reads ± S.D. = 73,017.25 ± 33,996.54 across both teams and genes, Figure 2, Supplementary S1 tab “data for analysis”), which contained non-sealed products that were most likely subjected to significant environmental contamination from sawdust and soil in the pallets. Compared to pallet B3, there was approximately 500 times less reads for *R. pseudoacacia* detected in samples from pallets B7 (72.63 ± 176.86) and B9 (86.18 ± 193.44) (Supplementary S1). In the same way, the field negative control showed 11 times fewer reads for *R. pseudoacacia* compared to pallet B3 and 3–5 times fewer reads compared to background negative controls from each pallet (FN_ITS2_ = 27,111 reads, Supplementary S1), suggesting that the contamination of DNA did not originate from the cold storage of the site, but from the pallets themselves. *Eucommia ulmoides* was also detected in pallets B3 (1036 ± 932.09) and B9 (2044 reads, single detection by PIC, Supplementary S1, tabs “data for analysis” and “data summary”) and field background negative controls (BC_B3 = 332.25 ± 105.71, BC_B7= 77 ± 63.11 and BC_B9 = 7538 ± 7835.57) across both teams and genes (Figure 2, Supplementary S1), albeit to a lesser degree compared to *R. pseudoacacia*. All pallets showed the presence of bacteria and fungi DNA (e.g., *Alternaria alternata*, *Penicilium* sp., *Murispora* sp., and *Mucor racemosus*), which were removed from further analyses (Supplementary S1, tab “data for analysis”).

### 2.3. Species Detection for the ITS2 Region

**Figure 3:**
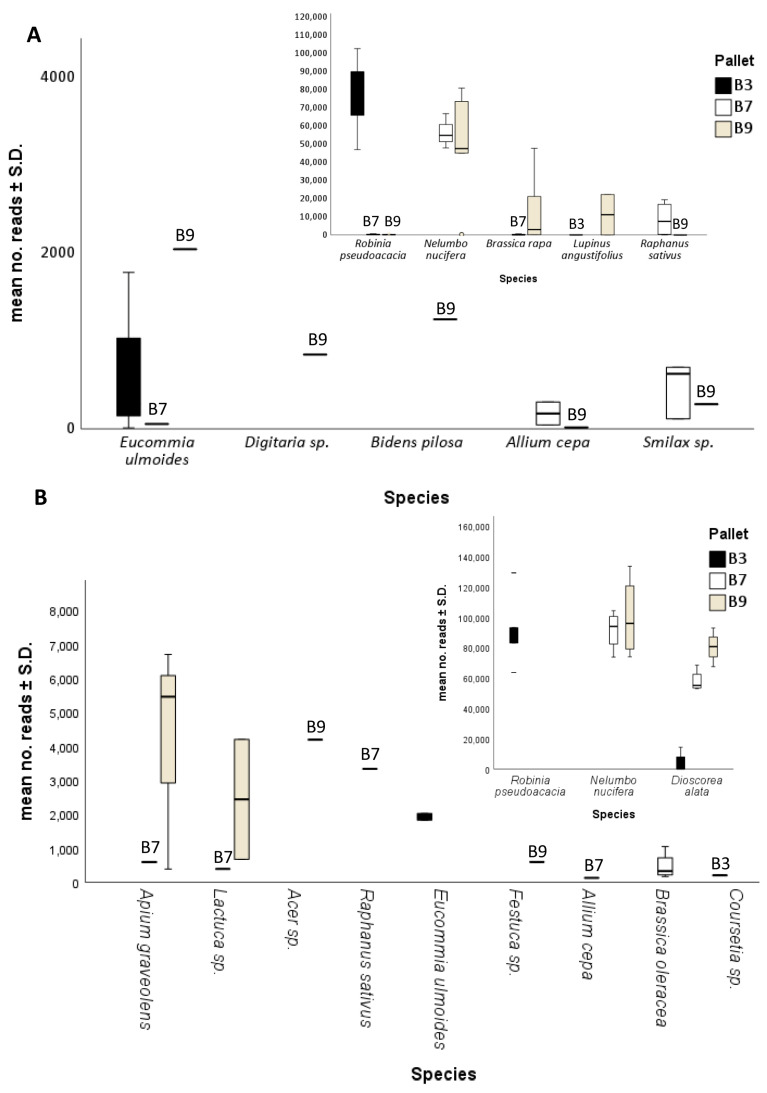
Species detection across both teams based on the mean number of reads ± S.D. for ITS2 (**A**) and *trnL* (**B**) gene regions across both teams. Inlays show detections for detections with >4000 and >8000 reads of ITS2 and *trnL*, respectively.

A total of seven species and three genera were detected by targeting the ITS2 region (Figure 3A). Pallet B3 showed high positive detections for *R. pseudoacacia* (78,227.13 ± 18,077.11) and *Eucommia ulmoides* (295 ± 573.88), both of which were detected across all field negative controls (Figure 2 and Figure 3A). Pallet B7 showed positive detections for five species (*A. cepa*, *E. ulmeoides*, *Nelumbo nucifera*, *Raphanus sativus*, and *R. pseudoacacia*) and two genera (*Brassica* sp. and *Smilax* sp.), of which the highest detections were for *N. nucifera* (21,234.5 ± 29,749.69) and *R. sativus* (4,313.38 ± 7880.73) (Figure 3A). Lastly, seven species (*A. cepa*, *Bidens Pilosa*, *E. ulmeoides*, *Lupinus angustifolius*, *Nelumbo nucifera*, *Raphanus sativus*, and *R. pseudoacacia*) and three genera (*Brassica* sp., *Digitaria* sp., and *Smilax* sp.) were detected in pallet B9, of which *N. nucifera* (30,974.88 ± 35,095.56), *Brassica rapa* (4313.38 ± 7880.73), and *L. angustifolius* (2795 ± 7893.34) showed the highest detections (Figure 3A). A tabular breakdown of the total number of species reads detected per gene sequenced and team by pallet and background control is available in Supplementary S1: Data summary.

### 2.4. Species Detection for the trnL Gene Region

A total of eight species and four genera were detected by targeting the *trnL* gene region (Figure 3B). As with the ITS2 region, pallet B3 showed high positive detections for *R. pseudoacacia* (67,807.38 ± 45,692.63), while positive detections were also made for *Discorea alata* (2805.25 ± 5426.90), *E. ulmoides* (590 ± 803.86), and *Coursetia* sp. (103.5 ± 119.54) (Figure 3B). Pallet B7 showed positive detections for six species (*A. cepa*, *Apium graveolens*, *Brassica oleracea*, *D. alata*, *N. nucifera*, and *R. sativus*) and one genus (*Lactuca* sp.), of which the highest detections were for *N. nucifera* (45,780.88 ± 49,679.65) and *D. alata* (28,981.38 ± 31,337) (Figure 3B). Lastly, three species (*Apium graveolens*, *D. alata*, and *N. nucifera*) and three genera (*Acer* sp., *Festuca* sp., and *Lactuca* sp.) were detected in pallet B9, of which *N. nucifera* (49,978.13 ± 56,194.65) and *D. alata* (40,265.38 ± 43,582.09) showed the highest detections (Figure 3B). A tabular breakdown of the total number of species reads detected per gene sequenced and team by pallet and background control is available in Supplementary S1: Data summary.

### 2.5. Species Endemicity Assessment

All plant species detected in this study, with the exception of *E. ulmoides*, are currently recorded as either being established within Australia or as having a cosmopolitan distribution (Table 1). *Eucommia ulmoides* is currently unknown to occur in Australia in both wild and farmed populations across all cross-checked reference databases.

## 3. Discussion

Illegally imported goods present a substantial biosecurity threat, as they can introduce pests and diseases that may have catastrophic impacts on the environment, agricultural industries, and local communities [17]. In this study, we demonstrated the effectiveness of using an eDNA metabarcoding-assisted surveillance approach to provide strong evidence as to the offshore origin/s of one of Australia’s largest biosecurity detections [18]. Environmental DNA-based methods remain unregulated by the Australian government, and have no mandatory requirements, standards, or protocols required by the government for formal biosecurity applications. As such, data from this study were used as supplementary evidence from an exploratory standpoint to help inform decision making by the department, not as a tool to directly support a regulatory or enforcement response. Indeed, our eDNA analysis was one of multiple tools used to attest to the origin of illegal plant produce as part of the biosecurity response [18]. This study used multiple layers of quality measures and controls to improve the reliability and accuracy of molecular data following testing standards under ISO/IEC 17025:2017. We demonstrate how future applications could be implemented to inform biosecurity responses and indicate how methods could be recognized and regulated in the future.

This study targeted one chloroplast gene region (*trnL*) and one nuclear repetitive region (ITS2) to improve taxonomic identification accuracy. DNA barcoding is a powerful tool for species identification, yet its effectiveness is hindered by incomplete reference databases that limit accuracy in species resolution. Although global efforts, such as the Barcode of Life Data System (BOLD), have expanded DNA libraries, many taxa remain underrepresented. Even well-documented groups like fishes and insects still lack complete barcode coverage, increasing the risk of misidentifications [19]. Targeting different regions to inform species identification improves how comprehensive taxonomic identity steps can be in metabarcoding workflows, providing redundancies in data comparison of both markers against reference databases. Other studies have harnessed the power of these markers to effectively trace the origins of goods. For example, the use of the *trnL-trnF*, ITS2, and other molecular loci have been proposed to determine the species origin of agarwood traded in the market [20]. Similarly, the use of the ITS2 locus has also been used to determine the authenticity and geographical origin of bee honey [11]. Both *trnL* (UAA) and ITS2 markers proved invaluable in this study by revealing the plant species composition, even in degraded samples. We recommend that future applications of eDNA-based metabarcoding for the purpose of informing biosecurity decision making target multiple genetic markers to improve taxonomic resolution and accuracy in identification.

Amplification of samples and controls was independently carried out in parallel by two research groups to strengthen the reliability of outcomes. The lack of standardized methodologies affects the reproducibility and comparability of results across studies. Variations in DNA extraction techniques, amplification protocols, and data analysis pipelines lead to inconsistencies [11,21], and the absence of uniform procedures makes it challenging to establish regulatory frameworks and validate DNA barcoding as a universal authentication tool. Moreover, previous research has demonstrated how eDNA extraction steps are prone to variability across testing facilities and minimize comparison capacity [5,22], highlighting the need to standardize DNA extraction, primers, and bioinformatic analyses for improved accuracy [23]. We followed these recommendations by having sample collection and extractions carried out by a single team (NeRC), and standardizing assays and bioinformatic pipelines, which provided greater certainty of the validity of results and a statistical framework for consistency. Significant differences in the number of ITS2 reads achieved by NeRC and PIC can be attributed to differences in sample normalization and amplification prior to sequencing, as a significant number of reads were lost during filtration and merger stages of reads produced by PIC. This loss of reads highlights the importance of high-quality standards for DNA normalization prior to qPCR analyses to ensure that DNA concentration and its associated concentration of inhibiting factors are adequately normalized to improve qPCR reactions and how many reads are retained throughout bioinformatic processing [22]. Parallel testing of samples could improve the uptake of eDNA-based applications in biosecurity by ensuring the authenticity and quality of laboratory testing practices.

Instances of detection congruency across teams in this study highlights the need for formal decision-making frameworks in biosecurity. In this study, there were nine instances where trace DNA for plant species was detected by one of the teams, which raises concerns on the proliferation of false positive results associated with the parallel testing of samples. Indeed, shipment declarations required by the government must contain an inventory with details of the produce and quantity present in the consignment, which could have been used to confirm and corroborate molecular data in this study. However, this consignment had multiple instances of regulatory nonconformance that included the incorrect provision of information within the shipment declaration associated with these confiscated goods. In the absence of transparent trade declarations commonly associated with suspicious consignments, as well as the absence of decision-making frameworks of HTS data in biosecurity applications, molecular results in this study must be taken as a composite of a much bigger picture rather than unique individual detections that could be confirmed by further tests and assessment of the produce. Moreover, these detections also had a significantly low number of reads associated with them when compared to all other detections achieved by both teams (Supplementary S1: Data summary), highlighting that low abundant species remain a source of inaccuracy in parallel testing of samples [5,22]. We recommend that future frameworks have a clear baseline for the number of reads associated with a detection to be accepted. In this study, we use a cut-off value of 100 reads; this however could be higher to minimize spurious detection of less abundant trace DNA.

This study highlights the importance of time and the impact of degradation on molecular workflows, and their capacity to inform biosecurity decision making. A total of 63 days had passed between the time of detection by biosecurity officers on the 15 February to the time of sample collection for this study on the 19 April 2023. Significant degradation was observed across all pallets and produce, with evidence of multiple freeze–thaw cycles that are common to the continuous handling of perishable goods in suboptimal conditions. This was further demonstrated by the high detection of bacteria and fungi commonly associated with degraded food and plant products (i.e., *Alternaria alternata*, *Candida tetrigidarum*, *Rahnella inusitata*, *Stemphylium vesicarium*) across all pallets. Degradation significantly impacts the quality of trace DNA molecular workflows [24], making it difficult to recover suitable genetic material to reliably infer the presence of species within a sample, impacting on their detectability, confidence levels of identification, and biological relevance for species identification [25]. This is particularly relevant for metabarcoding studies dealing with highly processed products [26]. We have highlighted the importance of targeting multiple genetic loci to improve taxonomic coverage; here, we would also highlight the importance of considering timeframes within which an expected coverage and depth would be reliably achievable for biosecurity decision making, accounting for eDNA degradation. Not only would these timeframes allow for greater indication of which molecular techniques would be suitable for use, they would also improve confidence in detecting DNA in low abundance that would otherwise be undetectable due to degradation and higher backgrounds, and greater cost-effective value for future biosecurity applications considering the use of eDNA-based techniques.

The use of background and field controls was essential to understand the source of DNA across those pallets tested. The detection of ubiquitous fungal genera, such as *Alternaria*, *Penicillium*, *Murispora*, and *Mucor*, demonstrated the resolution power and sensitivity of the ITS2 locus to resolve the genera even in the highly complex eDNA matrix analysed in this study. Similarly, the significantly higher number of reads linked to *Robinia pseudoacacia* in background controls and pallet 3 (where products were packed with sawdust and soil and not vacuum-sealed) provided evidence to suggest that detections for the species were likely derived from the sawdust present in the boxes, rather than actual plant produce in the pallets, or from the cold room used to hold the pallets during the response. Similarly, detection of *Eucommia ulmoides* (a species not known to occur in Australia) across pallets and background controls, with its lack of detection in field negative controls from the cold room, along with data showing that no Australian native species were detected, provided strong evidence that plant products in these pallets were not packed in Australia and originated from another country.

DNA barcoding techniques have been previously used to support executive decisions in identifying illegal wildlife trade and managing biosecurity risks. For example, a consignment labelled as “fish meat” was intercepted at a Pakistani port. DNA barcoding revealed a 99% match with the endangered Indian flap-shelled turtle (*Lissemys punctata*), exposing the smuggling of protected turtles under false labelling [27]. Similarly, DNA barcoding has helped identify illegally imported plant-based products, aiding in trade regulation enforcement. For example, Salep, a traditional beverage made from orchid tubers in the Middle East and Europe, was shown to contain DNA from protected species, confirming illegal trade [28]. Lastly, DNA barcoding also ensures the authenticity of plant-based perishable goods, as is the case for species in herbal supplements, where species verification can reveal undeclared plant ingredients, underscoring its role in food safety and fraud prevention [29]. Numerous studies, including this one, demonstrate the capacity of molecular methods to complement biosecurity decision making.

Non-compliance with laws, trade restrictions, food safety standards, and health regulations amplify biosecurity risks [3]. Within Australia, the regulatory reform agenda ensures ongoing sta vc keholder engagement and a system of regulation that is conducive to ongoing improvements for assessing risk, compiling evidence, and reducing the time and cost of biosecurity management practices [30]. This compliance policy and enforcement framework supports the existing *Biosecurity Act 2015* legislation [31]. Application of molecular techniques can be used to support traceability and verify product claims. For black market goods, the origin of consignments is often made intentionally difficult to track through false documentation. Our results supported a body of evidence indicating suspicious trade of plant products, demonstrating the instrumental role of DNA barcoding for environmental DNA detection and identification for decision-makers.

## 4. Materials and Methods

### 4.1. Sample Collection

On the 17 February 2023, the Australian Government Department of Agriculture, Fisheries and Forestry confiscated ~38 tonnes of suspected illegal goods (SIGs) from shipping container imports deemed to be a potential biosecurity risk. These goods were subsequently quarantined at a shipping yard in Banksmeadow, New South Whales, Australia. Given the volume of the confiscated produce, pallets were individually wrapped in plastic film and segregated amongst unused shipping containers at room temperature, in empty cold rooms (4 °C), and in freezing storage rooms (−20 °C).

A subset of pallets was identified to maximise the chance of DNA capture from the origin of goods: specifically, those containing vacuum-sealed tuber products and quarantined at −20 °C. On the 19 April 2024, shipping yard officers transferred three pallets (B3, B7, and B9) into a large walk-in cold room for sampling by NeRC and departmental field officers. Prior to sampling, working surfaces were decontaminated, and officers wore full-body gowns and gloves during sample collection.

**Figure 4:**
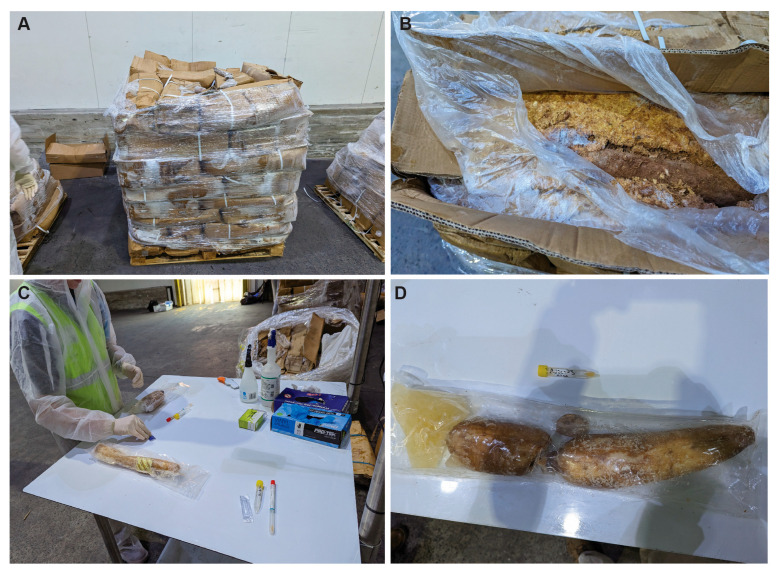
Photographs taken from one of the pallets before sampling (**A**), an example of unsealed tuber products in Pallet B3 (**B**), the processing of sealed tuber products, (**C**) and the condition of sealed tuber in pallets B7 and B9 (**D**).

After removing the plastic wrap surrounding each pallet, contents were visually inspected. Pallets contained multiple cardboard boxes with internal sawdust packaging surrounding partially frozen plant products. There was evidence of advanced degradation and decomposition at the time of sampling: fermented plant produce, and mould in the soil/sawdust packaging (Figure 4). One of the pallets (B3) contained unsealed vegetables loosely packed amongst the sawdust packaging. The other two pallets (B7 and B9) contained vacuum-sealed suspected illegal goods tuber produce).

Two background controls (BCs) were first collected from each pallet. One BC involved collecting ~2 g of sawdust/soil from the packaging materials inside the pallet into a 10 mL falcon tube with 8 mL of 80% ethanol. The soil/sawdust was collected from across multiple boxes within a pallet using sterile forceps. The other BC involved swabbing the exterior of boxes and bags within the pallet for ~30 s using a regular FLOQswab (COPAN Diagnostics). Both BCs per pallet were collected prior to collecting tuber swab samples.

A set of four samples (S) from each pallet was collected using a regular FLOQswab by swabbing the external surface of vegetable produce. For the pallet with loosely packed vegetable produce (B3), the surfaces of four different SIGs were swabbed for 30 s and preserved in 10 mL falcon tubes with 5 mL of 80% ethanol. For the pallets containing vacuum-sealed SIGs (B7 and B9), four different sealed SIGs were taken to a clean table and the outer layer of the sealed package was disinfected with bleach and thoroughly dried. The plastic packaging was then lacerated with a sterile scalpel blade, and the surface of the SIGs, as well as any soil particles, were swabbed for ~30 s using one swab. Each sample was collected sequentially and preserved in 10 mL falcon tubes with 5 mL of 80% ethanol. Post-sampling, two field controls (FCs) of the cold room were taken to determine possible airborne cross-contamination. These two controls were collected by opening 10 mL falcon tubes containing 5 mL of 80% ethanol and holding them near the pallets and around the cold room for ~1 min. All SIG samples (Ss), background controls (BCs), and field controls (FCs) were transported to the University of Canberra at room temperature immediately after sampling and stored at −20 °C on arrival.

### 4.2. DNA Extraction

Environmental DNA was extracted by NeRC at the University of Canberra using the DNeasy Plant Pro Kit (Cat. No 69204, Qiagen, Hilden, Germany). The quick-start protocol was modified to accommodate the different sample types’ (S, BC, FC) extraction material, as outlined below. All other steps were conducted as per the manufacturer’s protocol.

For S and BC samples, swab heads and sawdust/soil debris were lysed separately from the affiliated ethanol preservative. Swab heads and sawdust/soil debris were air-dried to limit residual ethanol carry-over prior to the addition of solution CD1. Affiliated ethanol was centrifuged at maximum speed for 15 min to pelletise particulates. The supernatant was aspirated off and discarded, avoiding the pellet. The pellet was then resuspended in 500 µL of solution CD1. They were later pooled by sample for binding onto a single spin column membrane to maximise yield. For FC samples, ethanol was centrifuged at maximum speed for 15 min to pelletise particulates. The supernatant was aspirated off and discarded, avoiding the pellet. The pellet was then resuspended in 500 µL of solution CD1. Six extraction controls (e-) were generated, each from 500 µL of solution CD1 in tissue disruption tubes kept open in the extraction hood for ~20 s.

Incubation at 65 °C occurred in a hybridisation oven (model HO35, Ratek, Boronia, Australia) with a rocking platform (HO35RP, Ratek, Boronia, Australia). High-speed centrifugation steps were performed with a high-speed centrifuge (model 5430R, Eppendorf, Hamburg, Germany), and brief spins were performed with a mini-centrifuge (model D1008, DLAB, Beijing, China). Vortexing steps were performed using a multi-tube mixer (model MTV1, Ratek, Boronia, Australia). The DNA was eluted in 80 µL of UltraPure™ DNase/RNase-free dH_2_O (UPdH_2_O) (Cat. No 10977015, Invitrogen™, Carlsbad, CA, USA) and DNA purity and yield were assessed using a spectrophotometer (Nanodrop™ One^C^, ThermoFisher™, Wilmington, DE, USA) and gel electrophoresis. Lastly, 40 µL aliquots of each extract were shipped to the Plant Innovation Centre at the departmental post-entry quarantine facility in Mickleham, Victoria, (PIC) for parallel quantitative PCR amplification and purification of target amplicons.

### 4.3. Quantitative PCR Amplification

Two markers were selected for multi-locus metabarcoding to balance taxonomic resolution, discriminatory power, and preferential amplification of plant DNA, the Internal Transcribed Spacer 2 (ITS2), and the chloroplast trnL (UAA) P6 Loop (hereafter trnL) gene regions [16]. World-wide reference sequence databases are widely populated for seed plants for these two regions. Compared to other common plant metabarcoding targets such as Maturase K (matK) and ribulose 1,5-biophosphate carboxylase (rbcL), these shorter genetic markers and “mini-barcodes” are especially advantageous for eDNA and ancient DNA metabarcoding and to better compensate for degradation [9,10,11].

A 226 bp fragment of the ITS2 gene region was amplified using primers S2F (fwd) (5′-ATGCGATACTTGGTGTGAAT-3′) and 4 rev (rev) (5′-TCCTCCGCTTATTGATATGC-3′), while a 113 bp fragment of trnL was amplified using forward primers (5′-GGGCAATCCTGAGCCAA-3′) and reverse primers (5′-CCATTGAGTCTCTGCACCTATC-3′) [16]. Each qPCR reaction for each assay contained 3 µL of 5x MyFi™ Reaction Buffer (cat. no BIO-21117, MeridianBioscience^®^, London, UK), 0.3 µL of forward and reverse primers (both at 10 uM), 0.3 µL of MyFi™ DNA Polymerase (cat. no BIO-21117, MeridianBioscience^®^, London, UK), 0.4 µL of 5x SYBR Green (Cat. No. S7563, Invitrogen™,Carlsbad, CA, USA), 8.5 µL of DNase/RNase-free UltraPure™ H_2_O and 2.5 µL of DNA extract, for a total reaction of 15 µL. Samples (S, BC, and FC) were analysed in triplicate together with their corresponding extraction negative controls, non-template qPCR controls, and a [10^5^ copies/µL template] synthetic double-stranded DNA positive control (gBlocks™ Gene Fragments, Integrated DNA Technologies, Singapore) (Table 2).

PCR conditions for ITS2 involved an initialisation step at 95 °C for 3 min followed by 45 cycles of 95 °C for 30 s, 55 °C for 30 s, 72 °C for 15 s, one extension phase at 72 °C for 1 min, and a final dissociation curve step of 95 °C for 15 s, 60 °C for 1 min, and 95 °C for 1 s at a ramp speed of 0.65 °C/s. PCR conditions for *trnL* were as above except that no extension step was used within the PCR cycles or as a phase. Quantitative PCR was conducted using an Applied Biosystems™ QuantStudio™ 7 Pro System (by NeRC) and a Bio-Rad Thermocycler CFX Opus 96 system (by PIC). Results were analysed with Design & Analysis 2.6.0 software (ThermoFisher Scientific, Waltham, MA, USA).

Three-step serial dilutions of each extract were tested by NeRC to assess sample inhibition and select the appropriate dilution for qPCR. Amplification curves and melt curves were used to assess amplification quality. Any sample or control replicates exhibiting no amplification, or displaying abnormal/non-target melt curve profiles, were assigned a non-detection status and omitted from further downstream processing.

### 4.4. Amplicon Purification

Amplicons for ITS2 produced by NeRC were purified using an AMPure XP (Ref: A63880, Beckman Coulter, Brea, CA, USA) bead protocol, following the manufacturer’s instructions. A 1.2x bead ratio was utilised, and purified product was eluted in 30 µL elution buffer. Amplicons produced for *trnL* by the UC-NeRC were excised and purified using a Purelink™ Quick Gel Extraction Kit (Cat. no K210012, Invitrogen™, Vilnius, Lithuania) following the manufacturer’s instructions. The optional isopropanol wash step to increase yield was conducted with 700 µL of Wash Buffer (W1), and purified amplicons were eluted in 30 µL DNase/RNase-free UltraPure dH_2_O. Similarly, amplicons produced by PIC using the *trnL* (UAA) P6 Loop assay were purified using QIAquick Nucleotide Removal Kit (Cat. No. 28306, Qiagen, Hilden, Germany) following the manufacturer’s instructions. Amplicons for ITS2 produced by PIC were purified following protocols using AMpure XP beads [16].

### 4.5. Library Preparation and Sequencing

Amplicons generated for each target region by NeRC and PIC were submitted to the Australian Genome Research Facility (AGRF) for library preparation and subsequent high-throughput sequencing. Separate libraries were prepared for each gene region using Nextera fusion primers with overhangs for barcoding and sequencing using an Illumina Miseq Reagent v2 (Cat. No MS-102-2003, Illumina, Singapore) kit (500 cycles, paired-end 250 bp reads).

### 4.6. Bioinformatic and Statistical Analysis

Fastq.gz files provided by AGRF were automatically demultiplexed using the Illumina Local Run Manager software wherein primers and adapters were trimmed from all reads. Following this, demultiplexed files were quality-evaluated, denoised, and filtered using DADA2 (1.22.0) [32]. Forward and reverse reads were truncated to 226 bp for ITS2 and 113 bp for trnL, a maximum number of expected errors = 2, and chimeric sequence removal was completed by consensus. Following this, multiple sequence alignment of Amplicon Sequence Variants (ASVs) was performed using MAFFT (EMBL’s European Bioinformatic Institute, Cambrideshire, UK) [33] and taxonomic information was assigned to each ASV against the nucleotide reference database of the National Center for Biotechnology Information (NCBI) using rBLAST (0.99.2) [34]. All assignments were completed using >95% pairwise similarity and >95% coverage against NCBI nucleotide accessions. ASVs and their associated reads were then grouped based on final taxonomic assignment into Operational Taxonomic Units (OTUs). Any OTU with less than 100 reads across all samples was removed from the study. All statistical analyses were conducted using SPSS (version 29.0.0.0, IBM, Corp., Armonk, NY, USA). Lastly, the status of detected plant species within Australia was cross-checked against reference databases maintained by The Atlas of Living Australia [35] and the Australian Virtual Herbarium [36] to confirm if species were currently considered ‘Established’ (i.e., wild or framed populations are known to occur in Australia), ‘Cosmopolitan’ (i.e., species that is globally distributed with wild and farmed populations reported in Australia and overseas), ‘Native’ (i.e., species that is endemic to Australia; wild and farmed populations are known to occur in Australia), or ‘unknown’ (i.e., no current wild or farmed populations are reported in Australia).

## 5. Conclusions

This research supports the use of environmental molecular techniques as a complementary tool in biosecurity applications. We demonstrate how the testing of environmental samples for trace DNA can be achieved across different teams to provide greater inference of molecular data in biosecurity applications, and how value can be provided to determine the origin of suspect products using high-throughput sequencing techniques. We provide considerations for targeted marker selection, preferential sampling of securely sealed goods, and the inclusion of controls to mitigate risk when working with degraded environmental samples and aerosolized DNA. Sequencing results from two groups (NeRC and PIC) operating in parallel for amplicon generation provide important information on the variation, congruence, and repeatability of molecular workflows under circumstances with limited deviation in processing methodology. Our methods provide important details for the adoption of standardized protocols in biosecurity applications and indicate where future applications can greatly improve methods and frameworks to improve confidence in the utility of molecular testing for biosecurity responses.

## Figures and Tables

**Table 1:** Endemicity assessment of species was based on national references from the Australian National Herbarium, The Atlas of Living Australia, and the Australian Virtual Herbarium. Species were considered ‘Established’ (i.e., wild or farmed populations are known to occur in Australia), ‘Cosmopolitan’ (i.e., species is globally distributed with wild and farmed populations reported in Australia and overseas), or ‘unknown’ (i.e., no current wild or farmed populations are reported in Australia). Native species (i.e., species endemic to Australia; wild and farmed populations are known to occur in Australia) were not detected in this study.

Species	No. of ASVs	No. Reads	Gene	Status in Australia	Endemicity
*Nelumbo nucifera*	271	1,183,747	ITS2 and *trnL*	Established	Asia–North Australia
*Robinia pseudoacacia*	351	1,170,817	ITS2 and *trnL*	Established	America–USA
*Dioscorea alata*	53	576,416	*trnL*	Established	Asia
*Brassica rapa*	12	75,633	ITS2 and *trnL*	Cosmopolitan	Scandinavia–Eastern Europe
*Raphanus sativus*	10	37,862	ITS2 and *trnL*	Established	Asia
*Lupinus angustifolius*	5	22,432	ITS2	Established	Europe–Asia
*Brassica napus*	16	21,007	ITS2	Cosmopolitan	Scandinavia–Eastern Europe
*Apium graveolens*	5	13,745	*trnL*	Cosmopolitan	Cosmopolitan
*Eucommia ulmoides*	18	8316	ITS2 and *trnL*	Unknown	Asia–China
*Brassica oleracea*	2	1320	*trnL*	Cosmopolitan	Scandinavia–Eastern Europe
*Bidens pilosa*	6	1246	ITS2	Established	America
*Allium cepa*	2	498	ITS2 and *trnL*	Cosmopolitan	Asia–mainland

**Table 2:** Synthetic dsDNA oligonucleotides used as qPCR positive controls in ITS2 and trnL (UAA) P6 Loop gene regions. Bolded sequences indicate primer binding sites.

Gene Region	Sequence 5′-3′ (Region Primers in Bold)	Fragment Length (bp)	Reference
ITS2	**ATGCGATACTTGGTGTGAAT** GAAAGCCTTAATTAATCAACAAATGTTTGGCTGCTAAAACCGCTTCCTTGAGTGTAGATGTTTCCACGCTGCCAGCCCACAAGGCGGAGTTGACCATCAGACGGCCGGATGAGCCACCCGTGTGGTCGACCTCGCTCGTCGCACAGCCTTCCTTGCCCTTGAAGGGGGTAAAGGTCCGGATAACCTTCCCCGTATGCCTTTTATGGAGACTGCCTGGCTCAGAGGGGATCCGGGGTCACGTTCGGGACCCGACTGTGGCAGCTGCTATGCCCTCCGCTTAGTCCCTGTATTTGGGTGTGCTGAATTGAGCGGAATACGGATCGGGCTAGTGGCTGCGCACGCCGCTCCAACTAACTATCGCCATTCTGAATGGCGC **AGCATATCAATAAGCGGAGGA**	417	[16]
*trnL* (UAA) P6 Loop	ATATTA**GGGCAATCCTGAGCCAA**AGACGAATACTCAAATACTACCACTGGAAGACAAAACATAGAGTAAGTAACCAAGGGAAAGAACCCATACACTA**GATAGGTGCAGAGACTCAATGG**TACTGA	125	[16], modified by UC-NeRC

## Data Availability

The original contributions presented in this study are included in the article/Supplementary Materials. Further inquiries can be directed to the corresponding author.

## Supplementary Materials

The following supporting information can be downloaded at https://www.mdpi.com/article/10.3390/ijms26115399/s1.

## References

[1] Michail N.A. (2020). World economic growth and seaborne trade volume: Quantifying the relationship. Transp. Res. Interdiscip. Perspect..

[2] United Nations (2022). UNCTAD Review of Maritime Transport 2022 Navigating Stormy Waters. 2022th ed.. https://unctad.org/rmt2022.

[3] García-Díaz P., Ross J.V., Woolnough A.P., Cassey P. (2017). The Illegal Wildlife Trade Is a Likely Source of Alien Species. Conserv. Lett..

[4] Smith K.F., Behrens M., Schloegel L.M., Marano N., Burgiel S., Daszak P. (2009). Reducing the Risks of the Wildlife Trade. Science.

[5] Zaiko A., Pochon X., Garcia-Vazquez E., Olenin S., Wood S.A. (2018). Advantages and Limitations of Environmental DNA/RNA Tools for Marine Biosecurity: Management and Surveillance of Non-indigenous Species. Front. Mar. Sci..

[6] Ferrier T., Casben L. (2023). Frog, Turtle Meat Seized in 38 Tonne Biosecurity Haul. AAP General News Wire. https://www.sheppnews.com.au/national/frog-turtle-meat-among-huge-biosecurity-hazard-haul/.

[7] Harris C. (2023). Box of Dead Turtles Among Tonnes of Products Found in Biosecurity Haul, in The Sydney Morning Herald. https://www.smh.com.au/national/nsw/box-of-dead-turtles-among-tonnes-of-products-found-in-biosecurity-haul-20230402-p5cxf4.html.

[8] De Brauwer M., Clarke L.J., Chariton A., Cooper M.K., De Bruyn M., Furlan E., MacDonald A.J., Rourke M.L., Sherman C.D.H., Suter L. (2023). Best practice guidelines for environmental DNA biomonitoring in Australia and New Zealand. Environ. DNA Hoboken N. J..

[9] Milla L., Sniderman K., Lines R., Mousavi-Derazmahalleh M., Encinas-Viso F. (2021). Pollen DNA metabarcoding identifies regional provenance and high plant diversity in Australian honey. Ecol. Evol..

[10] Pathiraja D., Cho J., Kim J., Choi I.-G. (2023). Metabarcoding of eDNA for tracking the floral and geographical origins of bee honey. Food Res. Int..

[11] Ullah S., Huyop F., Wahab R.A., Sujana I.G.A., Antara N.S., Gunam I.B.W. (2024). Using pollen DNA metabarcoding to trace the geographical and botanical origin of honey from Karangasem, Indonesia. Heliyon.

[12] Trujillo-González A., Thuo D.N., Divi U., Sparks K., Wallenius T., Gleeson D. (2022). Detection of Khapra Beetle Environmental DNA Using Portable Technologies in Australian Biosecurity. Front. Insect Sci..

[13] Bulman S.R., McDougal R.L., Hill K., Lear G. (2018). Opportunities and limitations for DNA metabarcoding in Australasian plant-pathogen biosecurity. Australas. Plant Pathol..

[14] Piper A.M., Batovska J., Cogan N.O.I., Weiss J., Cunningham J.P., Rodoni B.C., Blacket M.J. (2019). Prospects and challenges of implementing DNA metabarcoding for high-throughput insect surveillance. Gigascience.

[15] Taberlet P., Coissac E., Pompanon F., Gielly L., Miquel C., Valentini A., Vermat T., Corthier G., Brochmann C., Willerslev E. (2007). Power and limitations of the chloroplast trnL (UAA) intron for plant DNA barcoding. Nucleic Acids Res..

[16] Encinas-Viso F., Bovill J., Albrecht D.E., Florez-Fernandez J., Lessard B., Lumbers J., Rodriguez J., Schmidt-Lebuhn A., Zwick A., Milla L. (2023). Pollen DNA metabarcoding reveals cryptic diversity and high spatial turnover in alpine plant–pollinator networks. Mol. Ecol..

[17] DAFF (2019). Environmental Biosecurity Risk Management in Australia. Department of Agriculture, Fisheries and Forestry REVIEW REPORT NO. 2018–19/04. https://www.igb.gov.au/sites/default/files/documents/environmental-biosecurity-risk-management-australia.pdf.

[18] DAFF (2023). Minister Media Release: Operation Avoca Nets 38 Tonnes of Biosecurity Risks. https://minister.agriculture.gov.au/watt/media-releases/operation-avoca.

[19] Phillips J.D., Gillis D.J., Hanner R.H. (2019). Incomplete estimates of genetic diversity within species: Implications for DNA barcoding. Ecol. Evol..

[20] Lee S.Y., Ng W.L., Mahat M.N., Nazre M., Mohamed R. (2016). DNA Barcoding of the Endangered *Aquilaria* (Thymelaeaceae) and Its Application in Species Authentication of Agarwood Products Traded in the Market. PLoS ONE.

[21] Meyer C.P., Paulay G. (2005). DNA barcoding: Error rates based on comprehensive sampling. PLoS Biol..

[22] Phillips J.D., Gillis D.J., Hanner R.H. (2022). Lack of Statistical Rigor in DNA Barcoding Likely Invalidates the Presence of a True Species’ Barcode Gap. Front. Ecol. Evol..

[23] Rodriguez L.K., De Bonis L., McKee J., McKenna J.A., Urvois T., Barbaccia E., Dillane E., Lanfredi C., Hjellnes H., Jung A. (2025). Inter-laboratory ring test for environmental DNA extraction protocols: Implications for marine megafauna detection using three novel qPCR assays. Metabarcoding Metagenomics.

[24] Zaiko A., Greenfield P., Abbott C., Von Ammon U., Bilewitch J., Bunce M., Cristescu M.E., Chariton A., Dowle E., Geller J. (2022). Towards reproducible metabarcoding data: Lessons from an international cross-laboratory experiment. Mol. Ecol. Resour..

[25] Bowers H.A., Pochon X., Von Ammon U., Gemmell N., Stanton J.-A.L., Jeunen G.-J., Sherman C.D.H., Zaiko A. (2021). Towards the Optimization of eDNA/eRNA Sampling Technologies for Marine Biosecurity Surveillance. Water.

[26] Harrison J.B., Sunday J.M., Rogers S.M. (2019). Predicting the fate of eDNA in the environment and implications for studying biodiversity. Proc. R. Soc. Lond..

[27] Bruno A., Sandionigi A., Agostinetto G., Bernabovi L., Frigerio J., Casiraghi M., Labra M. (2019). Food Tracking Perspective: DNA Metabarcoding to Identify Plant Composition in Complex and Processed Food Products. Genes.

[28] Rehman A., Jafar S., Raja N.A., Mahar J. (2015). Use of DNA Barcoding to Control the Illegal Wildlife Trade: A CITES Case Report from Pakistan. J. Bioresour. Manag..

[29] De Boer H.J., Ghorbani A., Manzanilla V., Raclariu A.-C., Kreziou A., Ounjai S., Osathanunkul M., Gravendeel B. (2017). DNA metabarcoding of orchid-derived products reveals widespread illegal orchid trade. Proc. R. Soc. B.

[30] DAFF (2023). Regulatory Practice Statement. https://www.agriculture.gov.au/about/commitment/regulator-practice.

[31] DAFF Biosecurity Act: Biosecurity Act 2015. Federal Register of Legislation. Australian Government. https://www.legislation.gov.au/C2015A00061.

[32] Callahan B.J., McMurdie P.J., Rosen M.J., Han A.W., Johnson A.J.A., Holmes S.P. (2016). DADA2: High-resolution sample inference from Illumina amplicon data. Nat. Methods.

[33] Katoh M., Kuma M. (2002). MAFFT: A novel method for rapid multiple sequence alignment based on fast Fourier transform. Nucleic Acids Res..

[34] Hahsler M., Nagar A. (2024). rBLAST: R Interface for the Basic Local Alignment Search Tool.

[35] CSIRO (2025). Atlas of Living Australia. https://www.ala.org.au/.

[36] AVH (2025). The Australasian Virtual Herbarium. https://avh.chah.org.au.

